# The Beck Cognitive Insight Scale (BCIS): translation and validation of the Taiwanese version

**DOI:** 10.1186/1471-244X-10-27

**Published:** 2010-04-09

**Authors:** Yu-Chen Kao, Yia-Ping Liu

**Affiliations:** 1 Department of Psychiatry, SongShan Armed Forces General Hospital, Taipei, Taiwan; 2 Institute of Physiology, National Defense Medical Center, Taipei, Taiwan

## Abstract

**Background:**

Over the last few decades, research concerning the insight of patients with schizophrenia and its relationships with other clinical variables has been given much attention in the clinical setting. Since that time, a series of instruments assessing insight have been developed. The purpose of this study was to examine the reliability and validity of the Taiwanese version of the Beck Cognitive Insight Scale (BCIS). The BCIS is a self-administered instrument designed to evaluate cognitive processes that involves reevaluating patients' anomalous experiences and specific misinterpretations.

**Methods:**

The English language version of the BCIS was translated into Taiwanese for use in this study. A total of 180 subjects with and without psychosis completed the Taiwanese version of the BCIS and additional evaluations to assess researcher-rated insight scales and psychopathology. Psychometric properties (factor structures and various types of reliability and validity) were assessed for this translated questionnaire.

**Results:**

Overall, the Taiwanese version of the BCIS showed good reliability and stability over time. This translated scale comprised a two-factor solution corresponding to reflective attitude and certain attitude subscales. Following the validation of the internal structure of the scale, we obtained an R-C (reflective attitude minus certain attitude) index of the translated BCIS, representing the measurement of cognitive insight by subtracting the score of the certain attitude subscale from that of the reflective attitude subscale. As predicted, the differences in mean reflective attitude, certain attitude and R-C index between subjects with and without psychosis were significant. Our data also demonstrated that psychotic patients were significantly less reflective, more confident in their beliefs, and had less cognitive insight compared with nonpsychotic control groups.

**Conclusions:**

In light of these findings, we believe that the Taiwanese version of BCIS is a valid and reliable instrument for the assessment of cognitive insight in psychotic patients.

## Background

To fully understand the issues related to the clinical course of schizophrenia, patients' perspectives, beliefs, and values should be taken into consideration when assessing something as complex as insight. This will provide the clinician and researcher with a better understanding of the different models of psychotic illness, help-seeking, and mental health care acceptability [1,2]. Insight in psychiatric research has been regarded as a multi-dimensional construct that refers to awareness of illness-related issues, including symptoms of the illness, need for treatment, and consequences of the illness [2]. It is now well proven that schizophrenia is associated with a lack of insight [1-4], which can be profound and devastating [3,4]. Lack of insight is a matter of clinical concern because it has been associated with poorer adherence to medication and psychological treatment [5,6] and with social behavioural deficits [6], as well as with deficits in executive function [7,8].

Over the past few decades, researchers have focused their attention on the complex nature of insight [1,2]. Although clinicians have been measuring insight in psychotic patients for many years, there are still various problems and limitations associated with clinically-oriented insight scales. For instance, these clinically-oriented insight scales do not clarify the patients' limited capacity to access their anomalous experiences and misattributions [9]. The essential cognitive problem in schizophrenic patients centres not only on the consistent distortions of their experiences but also on their relative inability to distance themselves from these distortions and their relative impermeability to corrective feedback [9]. According to Beck et al. (2004), patients with psychosis may be impaired in their ability to examine and question beliefs and to interpret experiences, skills that they define as cognitive insight [9]. These studies point out that, in addition to consistently misinterpreting their reality, psychotic patients can not incorporate corrective feedback about their delusional beliefs [9,10]. They hypothesise that this impaired ability to question discordant information may contribute to the development and maintenance of delusional beliefs and thinking [9,10]. The Beck Cognitive Insight Scale was developed to assess this aspect of insight [9].

The initial study by Beck et al. found that the BCIS is composed of two subscales: self-reflectiveness and self-certainty [9]. The former includes items measuring objectivity, reflectiveness, and openness to feedback, and the latter measures certainty about one's own beliefs and judgments [9]. A composite index providing an estimate of overall cognitive insight is calculated by subtracting the score for the self-certainty subscale from the score for the self-reflectiveness subscale [9]. Reliability and validity of this insight scale have been demonstrated in a mixed group of inpatients with psychosis and depression [9], a group of middle-aged and older outpatients with schizophrenia [10], and a group of patients with bipolar disorder [11]. The BCIS has also been applied to non-clinical populations [12,13]. The internal consistency of BCIS is similar between clinical and non-clinical samples [11,13].

The majority of studies that have investigated the relationship between overall cognitive insight of schizophrenic patients as measured by the composite index scale of the BCIS and clinical insight as measured by the Scale to Assess Unawareness of Mental Disorder (SUMD) [9,14] and the Birchwood Insight Scale (IS) [10] have found that these variables are significantly related. For example, Beck et al. reported a correlation between SUMD awareness of delusion and self-reflectiveness, but no other correlation was found between mental illness and the composite index [9]. Pedrelli et al. observed a correlation between the self-reflectiveness and the relabel subscales and the total IS scale score [10].

To our knowledge, no similar instrument has been published and validated in the Taiwanese language. Consequently, the goal of our study was to describe the reliability and validity of the Taiwanese version of the BCIS originally developed by Beck et al. [9]. Participants were asked to rate the extent to which they agreed with each statement by using a 4-point scale ranging from 0, "do not agree at all", to 3, "agree completely." We propose that cognitive insight is a higher-level form of cognitive processing (metacognitions) that includes one's ability to distance oneself from one's misinterpretations and reappraise them [9]. In addition, insight scales' evaluations of these aspects tend to rely on the discrepancies between the views of clinicians and those of patients, thereby introducing further complexities to the phenomenon of insight that is elicited [10,15]. As discussed earlier, the BCIS reliably elicits patients' reports of their objectivity and receptiveness. The present study is a preliminary study that investigates whether the association of cognitive insight with psychopathology, clinical variables, and researcher-rated insight assessments found in previous research can be replicated in a Taiwanese context. Previous research has demonstrated that individuals with psychotic disorders have impaired self-reflectiveness and are overconfident relative to those without psychotic disorders [9]. We expect, then, that similar results will be demonstrated for those individuals who are psychosis prone in the present study.

## Methods

### Translation

The repeated forward-backward translation procedure was applied to translate the BCIS from English into Taiwanese language. One clinical psychologist and one psychiatrist translated the questionnaire into Taiwanese and two professional translators backward translated the Taiwanese into English. Any inconsistencies were resolved by retaining only the translated items that perfectly matched the original BCIS after back-translating the items into English. Subsequently, a provisional version of the Taiwanese questionnaire was developed, and a pilot study was performed with ten respondents with and without schizophrenia. Small revisions have been made to the translated version as a result of the pilot study's findings. Ultimately, a final Taiwanese version of the BCIS was used in this study.

### Participants

A cross-sectional study using the translated BCIS was conducted across three subgroups of study subjects. Group 1 consisted of 60 health control subjects (30 males and 30 females), including practical nursing students and staff members at a general hospital with no history of psychiatric disorders, who served as a general population comparison group. Group 2 comprised 60 patients (30 males and 30 females) with the diagnosis of major depressive disorder (MDD) without psychotic features, single episode or recurrent, and were outpatients who had been referred by the psychiatric department of a general hospital. Group 3 included 60 outpatients (31 males and 29 females) with the diagnosis of schizophrenia or schizoaffective disorder; the patients were recruited from the outpatient department of a general hospital. All of the diagnoses in our sample were made according to DSM-IV criteria [16] by a responsible trained psychiatrist. All patients had not been hospitalised during the previous six months. Small changes had been made to the prescriptions of 24 subjects with or without schizophrenic disorders during the past six months; however, all patients were clinically judged to be stable enough to undergo the assessment by a responsible psychiatrist. Prior to commencing the study, ethical approval was obtained from the Institutional Review Board of Tri-service General Hospital, National Defense Medical Center in Taiwan. Following a comprehensive explanation of this study to the participants, informed consent was obtained from all of them. The participants also underwent a comprehensive screening and assessment. The clinical procedure used involved the administration of a structured clinical interview, a detailed medical history, and a physical examination. Patients who had evidence of organic brain pathology including cerebral tumour, epilepsy, systemic disease, history of cranial trauma, brain surgery, or history of substance abuse or dependence in the past or present were excluded from this study.

### Measures

The following assessments were administrated in a single session with reference to the respondent's behaviour and experience over the previous 12 months. To identify the test-retest reliability of the BCIS measure in this study, 30 subjects, including 10 from each diagnostic group, completed the BCIS again four weeks after the initial assessment. All 30 patients were closely followed up by the same investigator during the time between assessments, permitting a longer interval to complete the test-retest procedure.

To assess the convergent validity of the Taiwanese version of the BCIS, we evaluated how the BCIS results compared with clinicians' and researchers' assessments of insight among people with schizophrenia or schizoaffective disorder. The researchers first assessed Item G12, "judgment and insight," of the PANSS [17,18]. This item was scored on a 7-point Likert response scale. The G12 item provided a rating of the subject's awareness of his/her psychiatric symptoms, his/her need for treatment, and the consequences of his/her psychiatric illness. A second assessment was performed based on the first three items of the shortened version of the Scale to Assess Unawareness of Mental Disorder (SUMD)--a standardised scale on which ratings are made based on a direct interview with a patient [19]. Scores on this scale ranging from one to three for items that assess the subject's (a) awareness of the mental illness, (b) awareness of the effects of medication, and (c) awareness of the consequences of the mental illness were assigned. A score of one indicated that the subject was "aware"; two, "somewhat aware/unaware"; and three, "severely unaware." In order to increase the reliability of the assessment, the scores on the three SUMD items were summed to obtain the total SUMD score. This score represents a more relevant measure of insight. High scores on both the G12 item and the SUMD indicated less awareness of one's psychiatric illness. Participants were rated on the PANSS and SUMD prior to completing the Taiwanese version of the BCIS.

The PANSS was developed in an attempt to provide a more comprehensive assessment of the psychopathology of schizophrenic patients and is widely used in clinical and research settings; it is regarded as a reliable means of symptom assessment [17,18]. In the current study, all patients with psychosis were interviewed by a psychiatrist trained in the use of the PANSS, and five factor analytically-derived components PANSS were used, namely, positive, negative, cognitive, excited, and depressed.

### Statistical analysis

All statistical tests were carried out using the Statistical Package for the Social Science (SPSS) version 15.0 for Windows with the significance level set at P = 0.05 (two-tailed test).

### Validity of internal structure and reliability analyses

We conducted an exploratory principal components analysis (PCA) on the correlation matrix of the 15 items of the Taiwanese version of the BCIS. To clarify the interpretation, a varimax orthogonal rotation was employed. The exploratory approach of this study was justified by the extraction of factors with eigenvalues greater than or equal to 1.0. Construct validity and reliability were evaluated by calculating Cronbach's alpha coefficient for each factor.

### Correlation analysis

The score for each factor (the sum of the ratings for all items that constitute the factor) obtained using the present factor analysis and was utilised. Two researcher-rated insight scales, selected specific variables from the PANSS, and demographic and clinical characteristics were correlated with the analysed factor scores of the translated BCIS. Correlation analyses were performed using the Pearson coefficient when data were normally distributed; elsewhere, Spearman rank correlation was calculated.

### Statistical Analysis of Means

One-way analysis of variance (ANOVA) was used to test for differences between selected groups of BCIS subscales and index scores. To ensure that the BCIS subscales and index scores might differentiate patients (n = 60) with schizophrenia or schizoaffective disorder from patients (n = 60) with MDD without psychotic features and healthy controls (n = 60), independent t-tests were then performed to compare the mean BCIS subscale and index scores of patients with psychosis to those of subjects without psychosis. In considering the differences in the levels of education among the three selected groups, it should be noted that we attempted to statistically control for such differences. An analysis of covariance (ANCOVA) was performed to compare the three selected subgroups with level of education as a covariate (concomitant variable) that could influence the cognitive insight among the three studied groups.

## Results

### Subjects' characteristics

The demographic and clinical characteristics of the participants in the study are presented in Table 1. A total of 60 outpatients with schizophrenia and schizoaffective disorder, 60 outpatients with MDD without psychotic features, and 60 healthy controls participated in the study. The selected groups were well matched on all demographic and clinical variables, except for years of formal education. The data suggest that the psychotic patients (Group 3) had a significantly lower level of formal education.

**Table 1:** Demographic and clinical variables

	Schizophrenia (N = 60)	MDD (N = 60)	Health control (N = 60)	F/P
		
	Mean (SD)	Mean (SD)	Mean (SD)	
Age (years)	38.87 (9.19)	40.47 (14.45)	36.35 (10.03)	1.968 (0.143)
Education (years)	12.35 (2.64)	13.6 (2.72)	15.05 (2.01)	17.873**(< 0.001)
Duration of mental illness (years)	14.15 (8.21)	NA		
Onset of mental illness (years)	24.72 (7.74)	NA		
Number of previous hospitalisations	5.97 (3.74)	NA		
Gender (male/female)	31/29	30/30	30/30	
Antipsychotic agent (No/First/Second)	0/27/33	NA		
Mood stabilizer agent (No/Yes)	37/23	NA		
Hypnotic & Anxiolytic agent (No/Yes)	7/53	0/60		

**P < 0.01; MDD = Major depressive disorder; NA = non-available

### Validity of internal structure (construct validity) andreliability analyses

The results of the factor analysis indicated that the Kaiser-Meyer-Olkin measure of sampling adequacy was at an acceptable level of 0.72, and the Bartlett's test of sphericity was 483.89, P < 0.001, indicating that all of the correlations that were tested simultaneously were significantly different from zero. According to the principal components analysis (PCA) with varimax rotation, the first two eigenvalues were 4.24 and 2.66, accounting for 46.03% of the total variance. These eigenvalues indicated that two factors should be extracted and inspected for simple structure.

Each of the subscales was developed based on the factor loadings and applied in the subsequent analysis. For each item, the highest factor loading determined subscale inclusion. These two subscales can most suitably be described as the reflective attitude subscale and the certain attitude subscale (Table 2).

**Table 2:** Factor analysis and reliability coefficient

			BCIS-T^a)^		BCIS^b)^	
			**(n = 180)**		**(n = 150)**	
			**Factor**		**Factor**	

**Item**	**Statement**	**Attitude**	**I**	**II**	**I**	**II**

1	At times, I have misunderstood other's attitudes toward me.	R	0.50	0.06	0.58	0.07
2	My interpretations of my experiences are definitely right.	C	0.08	0.69	0.12	0.49
3	Other people can understand the cause of my unusual experiences better than I can.	R	0.60	0.10	0.43	0.11
4	I have jumped to conclusions too fast.	R	0.61	0.09	0.63	0.19
5	Some of my experiences that have seemed very real may have been due to my imagination.	R	0.76	-0.01	0.59	0.19
6	Some of the ideas I was certain were true turned out to be false.	R	0.62	-0.14	0.66	0.04
7	If something feels right, it means that it is right.	C	0.12	0.62	-0.06	0.64
8	Even though I feel strongly that I am right, I could be wrong.	R	0.31	0.25	0.57	-0.24
9	I know better than anyone else what my problems are	C	0.05	0.79	0.11	0.61
10	When people disagree with me, they are generally wrong.	R	0.49	0.20	0.08	0.67
11	I cannot trust other people's opinion about my experiences.	R	0.55	0.03	0.15	0.63
12	If somebody points out that my beliefs are wrong, I am willing to consider it.	C	-0.01	0.47	0.41	-0.1
13	I can trust my own judgments at all times.	C	0.07	0.69	-0.12	0.25
14	There is often more than one possible explanation for why people act the way they do.	C	0.04	0.55	0.33	-0.19
15	My unusual experiences may be due to my being extremely upset or stressed.	R	0.34	0.18	0.5	0.18
	% of Variance		28.3	17.7	18	14
	Cronbach's alpha coefficient		0.7	0.72	0.68	0.6

Note: Extraction with Rotation method: principal component analysis with Varimax

R = Reflective attitude subscale; C = Certain attitude subscale

a): Translated Taiwanese version of the Beck Cognitive Insight Scale, administered to native Taiwanese speakers.

b): The original Beck Cognitive Insight Scale, administered to native English speakers.

Based on concepts regarding self-correction derived from previous studies [20-22], it was hypothesised that the patients' level of certainty and resistance to correction of their beliefs might diminish their ability or willingness to be introspective, and the reflectiveness-certainty index would reflect this dampening of self-reflectiveness. Therefore, a R-C index was calculated (i.e., reflective attitude minus certain attitude) as the measure of cognitive insight in our study.

An internal consistency analysis was conducted on each of the two subscales. The reliabilities (coefficient alpha) of the two subscales of the translated BCIS for the 180 subjects were 0.7 for the reflective attitude subscale and 0.72 for the certain attitude subscale. Test-retest reliability was determined using the assessments of the 30 patients who repeated the BCIS after four weeks. The test-retest reliability coefficient over a four-week interval ranged from 0.75 to 0.79 at the subscales and R-C index level. (all P < 0.01). Given the results obtained, these two subscales were considered acceptable for the purpose of the research [23,24]. For the Taiwanese version of the BCIS, the alpha coefficients for the reflective attitude and certain attitude were 0.72 and 0.78, respectively, for the 60 (33.33%) outpatients with schizophrenia or schizoaffective disorder; 0.42 and 0.60, respectively, for the 60 (33.33%) outpatients with major depressive disorders; and 0.73 and 0.69, respectively, for the 60 (33.33%) healthy controls. It should be noted, however, that the reflective attitude subscale was not significantly correlated with the certain attitude subscale in this study. Furthermore, within the extensive reliability analysis of the SUMD and PANSS conducted on the data of 60 outpatients with schizophrenia and schizoaffective disorder in this study, the alpha coefficient of 0.827 and 0.76, respectively, were also found to be reliable.

### The association of the BCIS subscale and index scores with two researcher-rated insight scales, demographic and clinical characteristics, and psychopathology

The correlation of the BCIS subscale scores with the psychosocial/clinical characteristics and psychopathology for the selected groups are presented in Table 3. The results indicate no significant correlation between the BCIS subscales and the psychosocial variables in healthy controls and subjects with major depressive disorder.

**Table 3:** Correlations of the BCIS subscales and index with demographic and clinical characteristics by selected groups

Item	RA	CA	R-C index
**Schizophrenia subjects**			
Gender (0 = male, 1 = female)	-0.199	0.155	-0.27*
Age (years)	-0.011	-0.032	0.009
Education (years)	0.176	0.093	0.102
Duration of mental illness (years)	-0.033	-0.136	0.052
Onset of mental illness(years)	0.022	0.106	-0.044
Number of previous hospitalizations	-0.115	-0.022	0.028
Antipsychotic agents (1 = first, 2 = second)	-0.148	0.04	-0.156
Anticholinergic agent (0 = no, 1 = yes)	0.182	0.062	0.126
Mood stabilizers (0 = no, 1 = yes)	-0.142	0.183	-0.236
Hypnotic, anxiolytic agent (0 = no, 1 = yes)	0.033	0.039	0.006

**MDD subjects**			
Gender (0 = male, 1 = female)	-0.089	0.113	-0.153
Age (years)	-0.076	0.062	-0.106
Education (years)	-0.198	0.002	-0.162

**Health control subjects**			
Gender (0 = male,1 = female)	-0.183	0.141	-0.248
Age (years)	0.135	0.179	0.015
Education (years)	-0.125	-0.115	-0.043

*P < 0.05; MDD = Major depressive disorder; RA = Reflective attitude subscale; CA = Certain attitude subscale.

In addition, an intercorrelation matrix was calculated for patients with schizophrenia or schizoaffective disorder. There was no significant correlation between the other BCIS subscales and the psychosocial variables except for a significant negative correlation between the R-C index and gender (r = -0.27, P < 0.05). The correlations of the BCIS subscale scores with use of particular medications did not reach statistical significance.

Pearson correlations between the BCIS and two researcher-rated clinical insight scales were independently examined to evaluate the validity of the translated BCIS. The BCIS was uncorrelated with the two researcher-rated insight scales, SUMD and G12 item of the PANSS. Nevertheless, we had to note that the awareness, consequence, and medication subscales as well as the total SUMD scale were significantly correlated with all of the psychopathology measures (r = 0.372 to 0.661, P < 0.01), except for the depression component of the PANSS. The two researcher-rated insight scales, namely the SUMD subscales and the G12 item of the PANSS, demonstrated positive significant correlations (r = 0.751 and 0.906, respectively) for the 60 schizophrenic or schizoaffective outpatients.

We examined whether correlations existed between subscales and index scores derived from the scale and positive, negative, cognitive, depressed, and excited components derived from the factor analysis studies of the PANSS. The BCIS R-C index and its subscale scores did not correlate significantly with the PANSS total score, positive, negative, depressed, and excited factors. The cognitive factor of the five-factor model of the PANSS was not significantly correlated with the BCIS R-C index and subscale scores.

### Comparison of means across subgroups

To assess the discriminative validity of the Taiwanese BCIS, we used ANOVA to compare the mean test scores of the BCIS subscales and composite index for the patients and controls. The results are presented in Table 4. Before discussing the level of education effect, certain facts that were manifested in the patients and controls are worth considering. First, the mean reflective attitude subscale score (mean = 11.08, SD = 4.13) of the subjects with schizophrenia was lower than those of the MDD and healthy control subjects. The difference in the reflective attitude subscale scores among the three groups of subjects was significant (F = 7.18; P < 0.01). Second, the mean certain attitude subscale (mean = 12.15, SD = 2.75) of the subjects with schizophrenia was higher than the mean certain attitude subscales of the MDD and healthy control subjects, but there was no significant difference between patients and controls on certain attitude subscales (F = 2.505; P = 0.085). Third, the mean R-C index (mean = -1.07; SD = 3.1) of the subjects with schizophrenia was lower than the mean composite index of the MDD and healthy control subjects. Moreover, there was a significant difference between patients and controls in the R-C index (F = 12.538; P < 0.01).

**Table 4:** One way ANOVA of the BCIS subscales and index for selected groups

	Schizophrenia^a)^ (N = 60)	MDD^b)^ (N = 60)	Health Control^c)^ (N = 60)	F(sig.)
		
	Mean (SD)	Mean (SD)	Mean (SD)	
RA	11.08 (4.13)	13.43 (3.38)	13.65 (4.72)	7.18**
CA	12.15 (2.75)	10.87 (2.98)	11.3 (3.77)	2.505
R-C index	-1.07 (3.10)	2.57 (4.17)	2.35 (4.41)	12.853**

**P < 0.01; ACOVA = Analysis of variance; RA = Reflective attitude subscale; CA = Certain attitude subscale.

a): Outpatients with schizophrenia and schizoaffective disorder.

b): Outpatients with major depressive disorder, without psychotic features, single or recurrent.

C): Health control subjects.

Using education as a covariate, the results of the ANCOVA were not comparable to the findings just mentioned. The difference in the reflective attitude subscale scores among the three groups of subjects was significant (F = 5.45; P = 0.021). However, there was no significant difference between patients and controls on the certain attitude subscale (F = 1.092; P = 0.297) and the R-C index (F = 1.961; P = 0.163). In view of our findings, we then consider the determinative confounding factor in the selected subgroups. The results, therefore, should be treated circumspectly.

**Figure 1:**
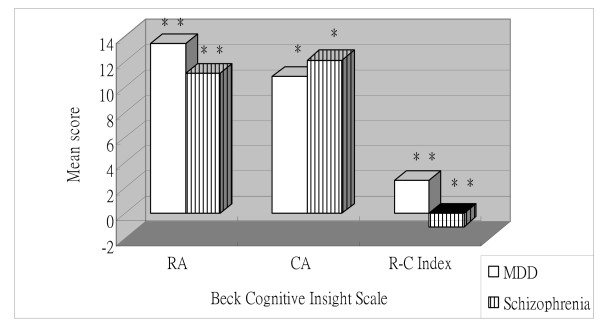
**Mean Beck Cognitive Insight subscale and index scores for subjects with schizophrenic disorders and with major depressive disorder (MDD)**. RA = Reflective attitude subscale; CA = Certain attitude subscale. **P < 0.01; *P < 0.05.

**Figure 2:**
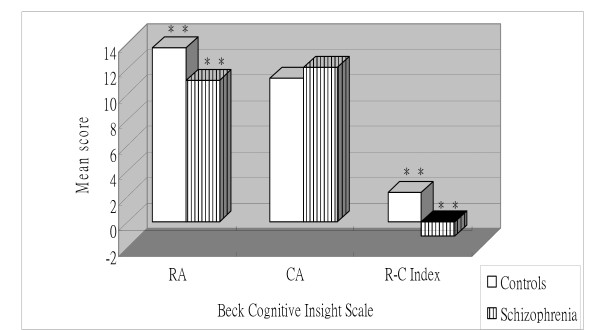
**Mean Beck Cognitive Insight subscale and index scores for subjects with schizophrenic disorders and controls**. RA = Reflective attitude subscale; CA = Certain attitude subscale. **P < 0.01.

**Figure 3:**
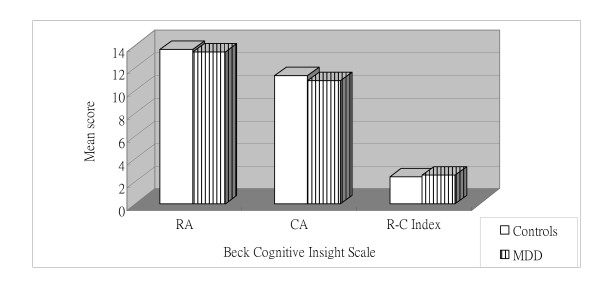
**Mean Beck Cognitive Insight subscale and index scores for subjects with major depressive disorder (MDD) and controls**. RA = Reflective attitude subscale; CA = Certain attitude subscale. (all P > 0.05).

Independent t-tests identified significant main effects of group for each of the two BCIS subscales and the index score. For the reflective attitude and R-C index scores, the patients diagnosed with schizophrenic disorders reported lower scores than subjects diagnosed with MDD and controls (P < 0.05) (Figure 1 and Figure 2). Additionally, the schizophrenic disorders group presented higher certain attitude scores than the MDD and control groups (P < 0.05) (Figure 1 and Figure 2). However, the MDD and control groups did not differ significantly from each other (Figure 3).

**Figure 4:**
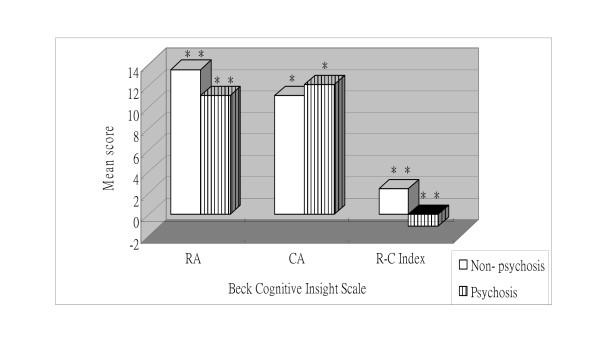
**Mean Beck Cognitive Insight subscale and index scores for subjects with and without psychotic disorders**. RA = Reflective attitude subscale; CA = Certain attitude subscale. **P < 0.01; *P < 0.05.

As shown in Figure 4, the mean reflective attitude score (mean = 11.08, SD = 4.13) of the patients with a psychotic diagnosis (n = 60) was lower than the mean reflective attitude score (mean = 13.54, SD = 4.09) of the subjects without psychosis (n = 120), t (178) = 3.788, P < 0.01. Furthermore, the mean self-certainty score (mean = 12.15, SD = 2.75) of the patients with a psychotic diagnosis was higher than the mean self-certainty score (mean = 11.08, SD = 3.39) of the subjects without psychosis, t (178) = -2.114, P < 0.05. Lastly, the mean R-C index score (mean = -1.06, SD = 4.62) of the patients with a psychotic diagnosis was lower than the mean R-C index score (mean = 2.45, SD = 4.28) of the subjects without psychosis, t (178) = 5.076, P < 0.01. In summary, the psychotic group demonstrated a pattern of lower reflective attitude scores, higher certain attitude scores, and lower R-C index scores compared to the non-psychotic groups.

## Discussion

The intent of this article was to develop and validate a cultural adaptation of the BCIS scale to measure Taiwanese schizophrenia patients' cognitive insight. The Taiwanese version of the BCIS, which is easy to administer in less than 15 minutes, proved to be acceptable to participants and clinicians, and its internal consistency and test-retest reliability were satisfactory. A R-C index of the BCIS was used to estimate a patient's level of cognitive insight.

There is general consensus that cognitive insight should be considered a multidimensional construct. This concept was also supported by our study. Exploratory factor analysis (EFA) of the Taiwanese version of the BCIS in the current study identified two factors that accounted for 46.03% of the variance, which resembled the two-factor structure of the original version. Additionally, note that these 15 items all had factor loadings higher than 0.3 in this study, which is consistent with previous studies [9]. However, the composition of the subjective insight domains as derived from factor analysis differed slightly from the initial theoretical model on which the scale was based. Our analysis revealed that item 10 ("When people disagree with me, they are generally wrong") and item 11 ("I cannot trust other people's opinion about my experience") were originally included in the self-certainty subscale of the previously reported BCIS [9] but had relatively larger factor loadings on the reflective attitude subscale in the present study. Similarly, item 12 ("If somebody points out that my beliefs are wrong, I am willing to consider it") and item 14 ("There is often more than one possible explanation for why people act the way they do"), which were originally included in the self-reflectiveness subscale of the previously reported BCIS [9], had relatively larger factor loadings on the certain attitude subscale in the present study. All 15 items of the Taiwanese version of the BCIS were distributed differently from the original structure of this insight scale after PCA, leading to a newly constructed instrument. These differences were not surprising because the questionnaire was based on the neuropsychological theoretical conception of cognitive insight, whereas factor analysis of the scale reflects the subjects' own perceptions of their cognitive insight. A partial explanation for the inconsistent results may lie in the fact that the selected items could not exactly measure what they were supposed to. In addition, their specificity might be imperfect as the scale refers to several overlapping dimensions. On the other hand, for the Taiwanese version of the BCIS, all items measuring reflective attitudes were strongly related to the first factor and all items measuring certain attitudes to the second factor. Thus, these factors can be accurately called reflective attitude and certain attitude, respectively.

The amounts of variance explained by the factors are somewhat different between the three cohorts in the present study and also found in previous studies [9,25]. The first factor explained 18% and the second factor 14% in an US sample [9], 16% and 12% in a Japanese sample [25], and 28% and 17% in the current study. Such dispersion might be derived from differences in cultural background or the origins and sizes of the samples.

For internal consistency, the reliability (Cronbach's alpha) in our study (reflective attitude subscale: alpha = 0.7, n = 180; certain attitude subscale: alpha = 0.72, n = 180) was higher than those in the original BCIS (all alpha<0.7) as reported by Beck et al. (2004) [9] and Pedrelli et al. (2004) [10], indicating that the Taiwanese version of the BCIS had more than adequate internal consistency in the current study and this scale could be used for individual clinical purposes. Furthermore, we found that the Cronbach's alpha of the certain attitude subscale was higher than that of the reflective attitude subscale in this study. It was somewhat surprising that the inverted Cronbach's alpha of the self-certainty subscale was substantially lower than that of the self-reflectiveness subscale in the previous studies [9,10]. Pedrelli et al. (2004) found that Cronbach's alpha for the entire measure was 0.66; for the self-reflectiveness scale, 0.7; and for the self-certainity subscale, 0.55, respectively [10].

Comparing the factor loadings of the original BCIS and the Taiwanese version, although the magnitudes of each factor were different, items measuring self-reflectiveness were gathered in the "reflective attitude" factor, and items of self-certainty were aggregated in the "certain attitude" factor for both subscales. In addition, the factor congruence coefficient indicated satisfactory factor agreement between the original and the Taiwanese version. These findings lead us to believe that that the original BCIS and the Taiwanese version are similar in their factor structures. Cronbach's alpha for each factor was high. This suggests that all factors were internally consistent. The equivalence between the BCIS and the translated BCIS was demonstrated through a similar factor structure and similar factor loading on particular items. However, as pointed out by Beck [9] and Pedrelli [10], the distinction between the two factors is irrelevant and the R-C index should be used for the purpose of measuring cognitive insight.

Although our principal components analysis yielded a two-factor solution, the observed pattern of intercorrelation supported the hypothesis that cognitive insight is not a unitary construct but one that comprises two or more related yet partially independent components. We hypothesised that patients' level of certainty about their beliefs might diminish their ability or willingness to be introspective and that the R-C index would reflect such a dampening of self-reflectiveness [9]. Therefore, the R-C index is interpreted as the measure of cognitive insight in the present study, as in the original BCIS [9]. The subscales of reflective attitude and certain attitude represent the following separate components of cognitive insight: (1) the patients' openness to feedback, recognition of having jumped to conclusions at times, and ability to acknowledge fallibility [25]; and (2) their overconfidence about belief or judgment [25], respectively. These descriptions suggest that subjects with psychosis are less self-reflective and more certain about their judgments than are subjects without psychosis. Warman et al. demonstrated that university students who had no history of psychotic disorders but were more prone to delusions were overconfident in their judgment [12], as are delusional patients with psychotic disorders [13]. Taken together, these findings show that cognitive insight may be evaluated quantitatively. The results of these studies can be useful for researchers studying insight in psychosis.

Our study also aimed to establish the convergent validity of the translated BCIS and its relationship with other traditional researcher-rated insight scales for insight evaluation in psychosis. The BCIS R-C index and its subscales did not significantly correlate with SUMD or the G12 item of the PANSS. The authors of the latter emphasise, in fact, that they are specifically examining attitudes towards illness and treatment. This contrasts markedly with previous studies' findings, which indicated moderate correlations between the R-C index score and the Birchwood Insight Scale relabel subscale score and total score [10]. One reason for these results could be that the insight phenomena captured by the different measures have different clinical/predictive values. To put it differently, different phenomena of insight are likely to be elicited in different situations and, indeed, the object of the insight assessment itself will have a determining influence on the actual phenomenon of insight that is elicited. In this study, the BCIS scale was designed to elicit a phenomenon of insight specific to the tendency of patients to question their knowledge and to be open to new information [10]. Therefore, it could be said that the BCIS described in this study was eliciting a different aspect of insight. Given the exploratory nature of these studies, any phenomenon of cognitive insight based on these preliminary finding should be treated with caution.

In fact, in our study, no correlation was found between the subscales and index score of the translated BCIS and the PANSS cognitive component which could also point to the fact that self-assessment of cognitive insight is independent of the clinical evaluation of cognitive functions. The correlation of cognitive insight with subjective perception of cognition in schizophrenia deserves to be considered and analysed.

It is, perhaps, unexpected that the BCIS subscales and index scores were not consistently related to positive and negative symptoms of the PANSS in this study. These finding are not in line with those of previous studies [10], in which the BCIS self-certainty factor correlated significantly with the PANSS total score and negative symptom and positive symptom factors, while the composite index correlated negatively with cognitive factors, indicating that increased insight was associated with fewer cognitive symptoms. However, these correlations were weak [10]. A possible explanation is that the items of the BCIS is designed to explore how patients face events in their lives, not restricted to unusual beliefs and events [12]. In other words, the BCIS does not exclusively assess judgments related to delusional beliefs [12]. Items are constructed to determine how individuals assess their judgments in general, not specifically in the context of unusual beliefs [12]. Although the association between the BCIS subscales and the PANSS was weak in our study, our findings support the BCIS's discriminative validity and show that a higher score on certain attitude and lower on reflective attitude and R-C index is a pattern specific to schizophrenic or schizoaffective patients. Inability to recognize when one is making an error or a tendency to be overly certain about one's interpretation of events might play a role in the emergence of psychosis.

Clinical insight is known to be associated with depression in patients with psychosis [26,27]. Patients with psychosis become depressed as insight increases [27]. It is not clear, however, whether a similar relationship exists with cognitive insight. Previous studies investigating this relationship have been reported conflicting results. Recently, one study found a correlation between depression measured by the Beck Depression Inventory-II (BDI-II) and cognitive insight in patients with schizophrenia or schizoaffective disorder [13], but another study did not find such a correlation [9]. Pedrelli et al. found no association between depression measured by the Hamilton Rating Scale for Depression and cognitive insight in middle-aged and older patients with schizophrenia or schizoaffective disorder [10]. In the present study, although it was not statistically significant, we found a correlation between the BCIS score and the depressed component of the PANSS suggesting that depressive symptoms in particular could affect self-assessment of cognitive insight by improving cognitive insight. Considering the influence of depressive traits on subjective perception of cognitive insight, we suggest measuring a patient's mood state when evaluating the cognitive insight.

A patient's understanding of his/her mental illness and its treatment is often influenced by his/her social and cultural background. A patient may have various culturally based beliefs that explain his/her illness and influence his/her coping strategies. Interestingly, a growing number of non-Western studies [28] that have examined the components of insight support its cross-cultural validity and the local adaptability of the assessment instruments [28]. Although there are considerable variations between our study and earlier published investigations [9,10,25] with regard to the mean subscales and the index score of the BCIS, some apparent and interesting similarities exist. It is interesting that when self-certainty was evaluated by the BCIS, those who had psychosis were more confident in their beliefs than those who did not. Similarly, the mean reflective attitude and the mean R-C index score of the outpatients with psychosis were lower than those of the subjects without psychosis. This aspect of cognitive insight appears to be, at least partially, a form of neurocognitive deficit or dysfunction that is somewhat independent of social and cultural influences. As an analogy, one would expect frontal or parietal dysfunction to disrupt self-awareness and other executive functions, regardless of ethnicity and cultural setting [29,30].

Like any study attempting to capture complex clinical realties, our study is limited in several ways. First, our sample was restricted, rather than employing randomly chosen subjects, and it consisted of mostly patients with chronic mental illnesses. It is possible that the results of this study may not generalise to all patients with psychosis outside of the selected group. Second, because the present study required informed consent and involved the psychopathological assessments, we did not include subjects who were very uncooperative. Because subjects who were very uncooperative were not included in the present study, demographic characteristics of non-volunteers are not available. However, it should be noted that uncooperative subjects were demographically different from the volunteers, so the generalisability of our result might be limited. Third, we readily acknowledge that our research was exploratory and that our recruitment procedure could be improved upon. Those participants who agreed to participate in the insight assessment may have had better relationships with the staff, may have more clearly perceived the beneficial effects of treatment, or may have had a higher insight level than those who did not. Fourth, in psychotic patients, cognitive deficit or dysfunction is probably the strongest predictor of insight assessment and future functional adaptability [31]. Determining reliable baseline cognitive function, particularly at the onset of the first episode of psychosis, may improve the predictive ability of these measures. However, in our study, insight assessments for patients with multiple relapses were limited due to the manifestations of neuropsychological deficits in psychotic disorders. Fifth, all of the psychotic outpatients who participated in our study were not naïve to antipsychotics. In fact, most of them took atypical antipsychotics, and none of them was drug-free at the time of assessment. It has been found that atypical antipsychotics improve some aspects of cognitive functioning [32]. However, the cognitive deficit can be either exacerbated or attenuated by concomitant antipsychotics, anticholinergics, or other agents, with an increasing confidence about some neurocognitive deficits in schizophrenia. Further research in this area would benefit from the investigation of the influence of medication effects and neuropsychological deficits on insight formation in psychosis. Sixth and lastly, the clinical ratings of cognitive deficits, such as the cognitive component of PANSS, were only a crude measure of the overall severity of cognitive dysfunction. Well-designed neuropsychological tests and more detailed evaluations of levels of cognitive disability are necessary in future studies of this sort.

## Conclusions

The results show that the Taiwanese version of the BCIS is a measurement instrument with adequate psychometric properties that can assess the impairments of cognitive insight present in patients with psychosis in research and clinical settings; this instrument can therefore improve the detection and prevention of these impairments. However, because the scale showed a two-factor structure that does not confirm perfectly to the theoretical basis for the BCIS, we intend to continue pursuing this line of investigation in a series of explorative studies. Accordingly, we recommend that the approach used in this study be replicated on larger and different populations.

## Competing interests

The authors declare that they have no competing interests.

## Authors' contributions

YCK wrote the draft of this manuscript. YCK and YPL conceptualised and designed the study. YCK collected and analysed the data. YPL supervised the study. YCK analysed the data further and wrote the final manuscript. YPL helped to revise the manuscript. All authors read and approved the paper.

## Pre-publication history

The pre-publication history for this paper can be accessed here:

http://www.biomedcentral.com/1471-244X/10/27/prepub
